# Exploring the therapeutic potential of targeting polycomb repressive complex 2 in lung cancer

**DOI:** 10.3389/fonc.2023.1216289

**Published:** 2023-10-16

**Authors:** Min Gao, Yongwen Li, Peijun Cao, Hongyu Liu, Jun Chen, Shirong Kang

**Affiliations:** ^1^ Department of Thoracic Surgery, The Affiliated Hospital of Inner Mongolia Medical University, Hohhot, China; ^2^ Inner Mongolia Medical University, First Clinical Medical College, Hohhot, China; ^3^ Tianjin Key Laboratory of Lung Cancer Metastasis and Tumor Microenvironment, Tianjin Lung Cancer Institute, Tianjin Medical University General Hospital, Tianjin, China; ^4^ Department of Lung Cancer Surgery, Tianjin Medical University General Hospital, Tianjin, China

**Keywords:** polycomb repressor complex 2, lung cancer, H3K27me3, EZH2, inhibitors

## Abstract

The pathogenesis of lung cancer (LC) is a multifaceted process that is influenced by a variety of factors. Alongside genetic mutations and environmental influences, there is increasing evidence that epigenetic mechanisms play a significant role in the development and progression of LC. The Polycomb repressive complex 2 (PRC2), composed of EZH1/2, SUZ12, and EED, is an epigenetic silencer that controls the expression of target genes and is crucial for cell identity in multicellular organisms. Abnormal expression of PRC2 has been shown to contribute to the progression of LC through several pathways. Although targeted inhibition of EZH2 has demonstrated potential in delaying the progression of LC and improving chemotherapy sensitivity, the effectiveness of enzymatic inhibitors of PRC2 in LC is limited, and a more comprehensive understanding of PRC2’s role is necessary. This paper reviews the core subunits of PRC2 and their interactions, and outlines the mechanisms of aberrant PRC2 expression in cancer and its role in tumor immunity. We also summarize the important role of PRC2 in regulating biological behaviors such as epithelial mesenchymal transition, invasive metastasis, apoptosis, cell cycle regulation, autophagy, and PRC2-mediated resistance to LC chemotherapeutic agents in LC cells. Lastly, we explored the latest breakthroughs in the research and evaluation of medications that target PRC2, as well as the latest findings from clinical studies investigating the efficacy of these drugs in the treatment of various human cancers.

## 1 Introduction

Lung cancer (LC) is one of the most common malignancies worldwide and has taken the lead role in cancer-related deaths in recent decades (1). Although progress has been made in early detection and molecularly targeted therapies, the prognosis for patients with advanced LC remains poor due to drug resistance and tumor recurrence and metastasis (2–4). As a result, identifying new therapeutic targets to offer personalized targeted therapy in precision medicine and overcome drug resistance has become a promising approach to improve treatment outcomes.

Epigenetics is a process that alters gene expression inheritance patterns without changing the DNA sequence. Epigenetic variations, such as DNA methylation, RNA modifications, and histone modifications, are more frequent and significant than somatic mutations and contribute to cancer development (5). Polycomb repressive complex 2 (PRC2) is a chromatin-modifying complex that is essential for maintaining cell differentiation and determining cell fate via the addition of lysine 27 trimethylation to histones (H3K27me3) (6, 7). Elevated levels of H3K27me3 in LC tissues are strongly associated with the development and prognosis of LC (8). Misexpression or mutation of PRC2 can lead to aberrations in cancer signaling pathways, contributing to cancer and other diseases (6). Due to its significant role in the development and progression of LC, PRC2 is a promising target for targeted therapies. The main objective of this article is to examine the association between PRC2 and the initiation, metastasis, and drug resistance of LC. It delves into the epigenetic factors involved in the development of lung cancer and reviews the most recent research developments in PRC2-targeted therapies for various types of tumors, particularly LC.

## 2 PRC2

**Figure 1:**
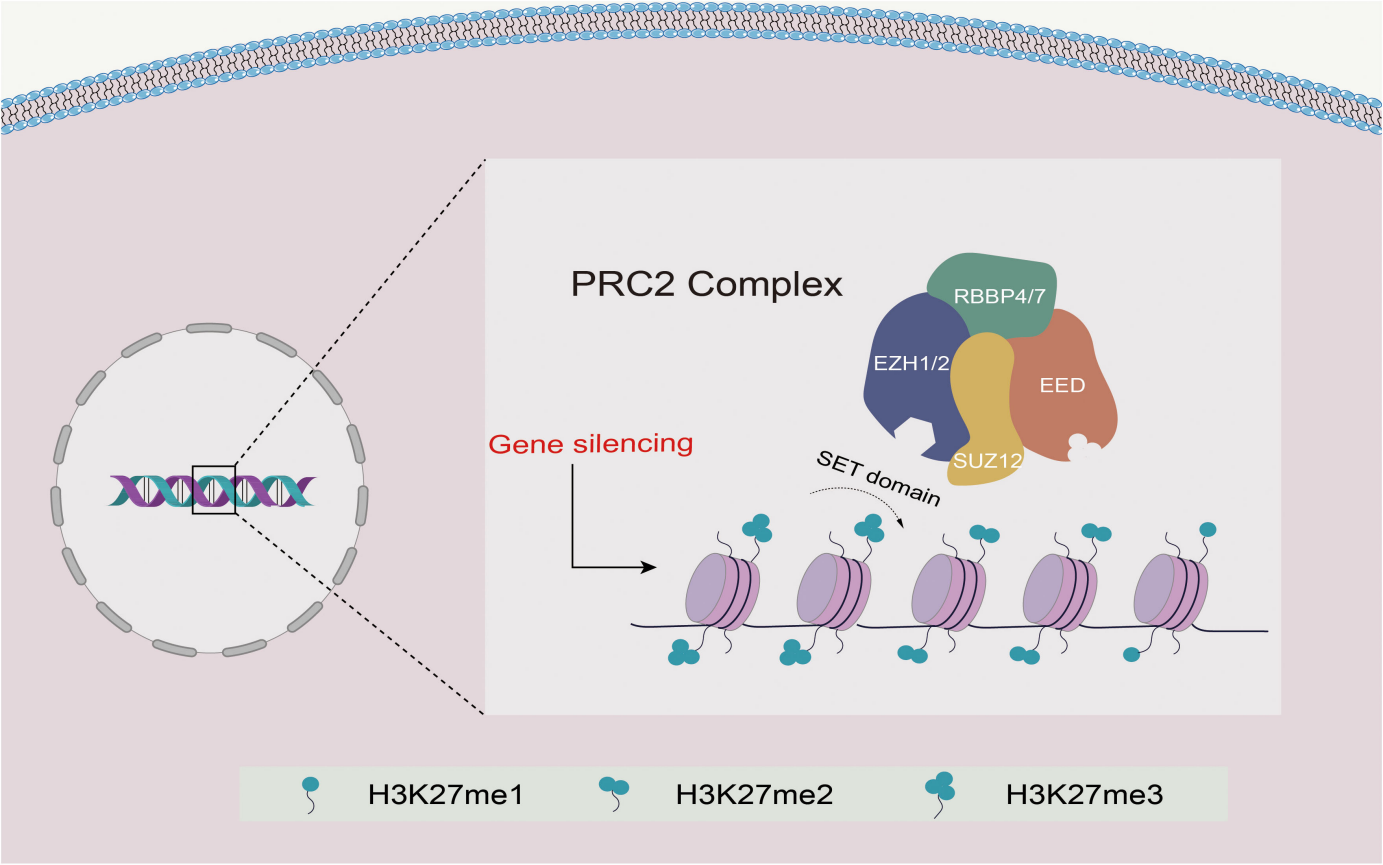
Schematic diagram of the structure and function of PRC2. EZH1/2 contains a SET domain with methyltransferase activity that mediates the methylation of H3K27 and promotes transcriptional silencing. EED binds to H3K27me3, resulting in allosteric activation of EZH1/2, while SUZ12 mainly stabilizes the complex. Together with RBBP4/7, they form the PRC2 complex, mediating PRC2 recruitment and promoting H3K27me1/2/3.

PRC2 possesses histone methyltransferase activity and is crucial for regulating cell differentiation and development. The catalytic roles of PRC2 are primarily played by the enhancer of zeste homolog 1 or 2 (EZH1/2), trimers of suppressor of zeste 12 protein homolog (SUZ12), and embryonic ectoderm development (EED) proteins, which interact interdependently to maintain the catalytic activity of PRC2 (9–13). These core subunits, along with retinoblastoma-binding protein 4 or 7 (RBBP4 and RBBP7; also known as RbAp48 and RbAp46), constitute the PRC2 complex responsible for all monomethylation, demethylation, and trimethylation (H3K27me1, H3K27me2, and H3K27me3) on histone H3, thus regulating gene expression by ensuring appropriate gene silencing (6, 14–16) (**Figure 1**).

## 3 PRC2 subunits and their interactions

The core subunits of PRC2 include SUZ12, EED, two methyltransferases (EZH1 and EZH2), RBBP4, and RBBP7. Both EZH1 and EZH2 exhibit enzymatic methyltransferase activity via the C-terminal SET domain (17). While EZH2 is the primary catalytic subunit that more efficiently methylates H3K27 than EZH1, EED and SUZ12 must also contribute to the catalytic activity of PRC2 to prevent autoinhibition of EZH2 (18–20). Following EZH2 trimethylates H3K27, the EED protein recognizes and binds to H3K27me3 through its WD40 domain, resulting in the activation of EZH2 in a positive feedback mechanism. This feedback loop allows the spread of H3K27me2/me3 modifications over large stretches of DNA, leading to the formation of extended domains of repression (21, 22). SUZ12 stabilizes PRC2 through its C-terminal VEFS domain, and its delta-VEFS can be recruited to target sites independently of other core subunits (15) (**Figure 1**).

Negative-stain electron microscopy has preliminarily investigated the conformation of PRC2 and the physical interactions among its subunits (17). PRC2 is composed of two lobes with distinct functions: the catalytic lobe and the targeting lobe, which are connected by SUZ12 (17, 23).

Within the catalytic leaf, the VEFS domain of SUZ12 interacts with either EZH1 or EZH2. By rotating its SET-inserted domain and rearranging the post-SET domain, the SET domain of EZH2 changes from an autoinhibitory to an active conformation that promotes its methyltransferase activity (12, 20, 24–26). The targeted lobe of PRC2 is composed of SUZ12, RBBP4/7, and several accessory proteins.

## 4 Mechanism of abnormal expression of PRC2 in cancer

### 4.1 PRC2 in transcription

PRC2 is a crucial epigenetic regulator involved in transcriptional repression and cell fate regulation in cancer development. The major catalytic subunit EZH2 is repressed at the transcriptional level through its SET domain, which functions as a histone methyltransferase to regulate transcription in a PRC2-dependent manner. EZH2 can form transcriptional complexes with other factors to activate target genes downstream. For example, SETD1A can induce H3K4me3 enrichment and activate transcription of NEAT1 and EZH2 in NSCLC, leading to activation of the Wnt/β-linked protein axis and promoting tumorigenesis (27).

There are several microRNAs that are known to play a role in the transcriptional regulation of PRC2 in LC. miR-4465 can bind to the 3’-UTR of EZH2, reducing its expression and inhibiting the proliferation, migration, and invasion of NSCLC cells (28). miR-145 can significantly reduce RBBP4 promoter activity, mRNA and protein levels, and hsa_circ_0102231 can promote NSCLC proliferation and invasion by reversing the anti-tumor effects of miR-145 through the miR-145/RBBP4 axis (29). Additionally, the functional conversion of EZH2 from histone methyltransferase to non-histone methyltransferase is dependent on long-stranded non-coding RNA (lncRNA) P21. Epigenetic silencing of the lncRNA SPRY4-IT1 in NSCLC cells occurs through direct binding of EZH2 to the SPRY4-IT1 promoter region, leading to transcriptional repression. The SPRY4-IT1 plays a role in the proliferation and metastasis of NSCLC cells by modulating epithelial-mesenchymal transition (EMT) through the regulation of e-calmodulin and waveform protein expression (30). In summary, PRC2 has diverse functions in controlling target gene expression through its interactions with various transcription factors and miRNAs and lncRNAs can influence the activity of PRC2 in lung cancer.

### 4.2 PRC2 in translation and post-translational

Post-translational modifications (PTMs) are essential biological processes in cancer development that regulate the conformation and functionality of proteins. EZH2, the primary catalytic subunit of PRC2, is subject to various PTMs, including phosphorylation, ubiquitination, O-GlcNAcylation, acetylation, and methylation. These PTMs can modulate EZH2 histone methyltransferase activity, localization, binding partners, and stability, affecting its role in cancer development and progression.

There is a growing body of evidence supporting the critical function of PTMs of EZH2 in cancer development. For example, EZH2 can be acetylated at lysine 348 (K348) by the acetyltransferase P300/CBP-associated factor (PCAF), which increases its stability, ability to repress target genes, and enhances the migration and invasion of LC cells (31). Phosphorylation of threonine 416 by cyclin-dependent kinase 2 promotes the recruitment of EZH2 at the target gene promoter, while methylation of lysine 307 (K307) by SMYD2 enhances the stability of EZH2 and promotes breast cancer cell proliferation, epithelial mesenchymal transition, and invasion (32, 33). O-GlcNAcylation of EZH2 at multiple sites, such as S73A, S76A, S84A, T313A, and S729A, enhances its stability and methylation enzyme activity, promoting tumor progression (34). In addition, cell cycle protein-dependent kinase 1 (CDK1)-mediated phosphorylation of threonines 345 and 487 promotes ubiquitinated degradation of EZH2 in breast cancer cells, cervical cancer cells, and LC cells (35). Investigating how PRC2 is regulated by various types of PTMs and its involvement in cancer development, as well as deciphering its underlying molecular mechanisms, may present promising opportunities for LC treatment.

### 4.3 PRC2 in cancer immunity

The tumor microenvironment (TME) is a complex immunoregulatory network in which various immune cells and factors interact with cancer cells. The expression of EZH2, the major subunit of the PRC2 complex, in both tumor and immune cells can induce epigenetic and transcriptomic changes, which mobilize various elements of the TME and promote the development of solid tumors with immunosuppressive activity.

EZH2 mediates T-cell activity in tumors primarily through the regulation of T-cell antigen-presenting genes, such as β-2-microglobulin (β2M), CTLA-4, the HLA family, and PD-L1. EZH2 can inhibit activation of T-cell by raising PD-L1 expression (36). Additionally, the effect of EZH2 on T-cell activity is dependent on various chemokines and cytokines within the TME, such as Th1-type chemokines like CXCL9 and CXCL10, which have a remarkable effect on the transport of effector CD8+ T cells. In ovarian tumors, EZH2 expression was negatively correlated with intratumoral CD8+ T cells, and its expression inhibited the production of CXCL9 and CXCL10, thereby affecting CD8+ T cell infiltration within the tumor (37, 38). Chemokine ligand 5 (CCL5) has also been identified as one of the key chemokines recruited by macrophages in tumor cells. CCL5 plays a huge part in the proliferation and invasion of LC cells (39, 40). Overexpression of EZH2 in LC cells promotes the production of tumor-derived CCL5, leading to recruitment of M2-like macrophages and immunosuppressive Treg cells, which can facilitate tumor proliferation and metastasis while inhibiting CD8+ T cells (41). T-cell exhaustion is defined as a low T-cell response and diminished function after prolonged antigen exposure. It is usually characterized by decreased production of cytokines, such as IFN-γ, TNF-α, and granzyme b, and is associated with poor clinical prognosis in solid tumors (42, 43). Inhibition of EZH2 induces a sustained antitumor response, culminating in T-cell failure and checkpoint activation (44).

Myeloid-derived suppressor cells (MDSCs) are a heterogeneous group of myeloid cells with immunosuppressive properties, derived from myeloid progenitors and immature myeloid cells. They play a huge part in cancer progression by shaping the tumor microenvironment through immunosuppression and inflammation (45). Loss of EZH2 activity is related to the production of MDSCs. Studies have shown that circulating-associated kinases activate the EZH2/NF-κB signaling pathway. This leads to the formation of a complex between EZH2 and NF-κB, which binds to the promoter of IL-6 and promotes the accumulation of immunosuppressive MDSCs (44). This, in turn, creates a microenvironment that suppresses the immune system and promotes drug resistance. EZH2 has been demonstrated to preserve the functionality of hematopoietic progenitor cells by stabilizing chromatin structure and suppressing the transcription of genes that promote differentiation (46). The EZH2 inhibitor GSK126 promotes the differentiation of hematopoietic progenitor cells into MDSCs, which can suppress CD4+ and CD8+ T cells, contributing to antitumor immunity (47).

Regulatory T cells (Tregs) are essential for immune homeostasis as they exert their effect by suppressing immune responses. However, their presence in tumor tissue impairs antitumor immunity and promotes tumor development and metastasis. EZH2 functions as an epigenetic silencer in tumor-infiltrating Treg cells to inhibit the expression of IL-4 and IFN-γ, while enhancing Foxp3 expression and Treg activation (48). Foxp3 recruits EZH2 to repress key genes during the inflammatory response in Treg cells (49–51). On the contrary, inhibition of EZH2 may impair Foxp3 expression, reduce the suppressive activity of Tregs, reshape the tumor microenvironment, enhance CD8 and CD4 effector T cell recruitment and function, and thus enhance antitumor immunity (52, 53).

Natural killer (NK) cells are essential suppressors of cancer process and are involved in the innate immune response (54).In the tumor microenvironment, NK cell differentiation and function are inhibited. The NK activation signaling pathway relies on the presence of NK group 2 member D (NKG2D) receptors on the cell membrane, and when the NKG2D receptor binds to its ligand, NK initiates a cytotoxic immune response in TME (55). EZH2 in cancer cells downregulates NKG2D ligand expression or TME and inhibits NK cell activation (36). EZH2 deletion or inhibition improves mature NK cell function and enhances its specificity by upregulating NKG2D (56). EZH2 inhibition enhances the eradication of hepatocellular carcinoma by NK cells in hepatocellular carcinoma (57). To summarize, modifying the epigenetic landscape can potentially improve the anti-tumor immune response by altering the tumor microenvironment. Therefore, combining immunotherapy with targeted inhibition of EZH2 represents a promising approach for cancer treatment.

## 5 Correlation of PRC2 with pulmonic carcinogenesis and invasive metastasis

**Figure 2:**
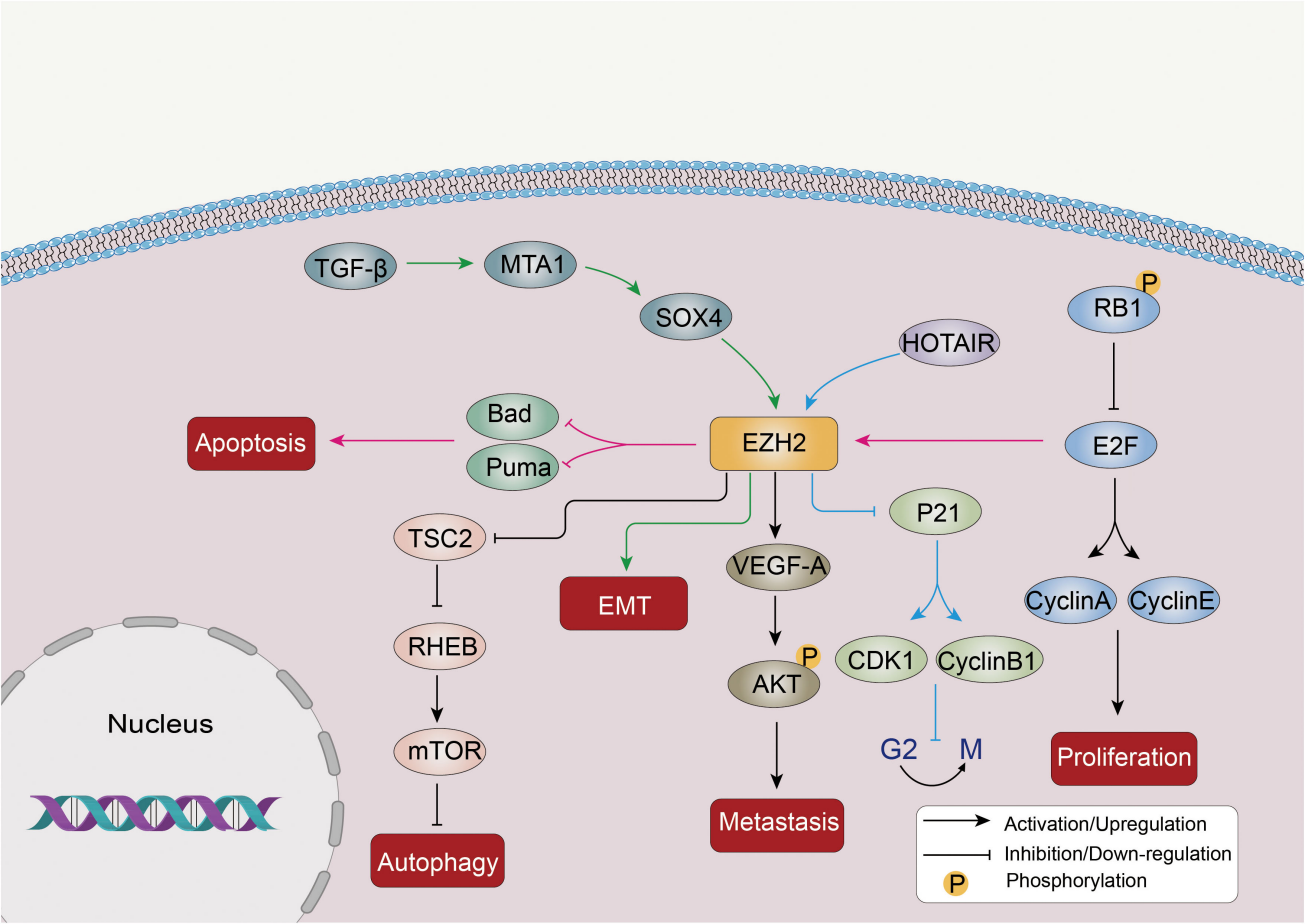
Abnormal expression of EZH2 promotes proliferation, invasion, metastasis and autophagy inhibition of LC cells through multiple mechanisms. EZH2 is involved in the EMT drive of the TGF-β-MTA1-SOX4-EZH2 signaling axis in cancer. In addition, EZH2 can inhibit TSC2, the negative regulator of the mTOR pathway, and further inhibit RHEB expression, thereby activating the mTOR signaling pathway and inhibiting autophagy. Other molecular pathways such as STAT3 and P21 are also affected by EZH2 in LC.

LC is a complex disease influenced by many factors, including genetics. In recent years, researchers have focused on the role of the PRC2 complex, specifically EZH2, in promoting the invasive metastasis of LC cells (**Figure 2**).

EZH2 promotes EMT, a process in which epithelial cells acquire a mesenchymal phenotype. EMT is related to many cancer features, including metastasis, immune evasion, and resistance to therapy (58). SOX4 activates EMT by directly binding to the EZH2 promoter and upregulating its expression (59). TGF-β induces the downstream targets MTA1 and SOX4, leading to further activation of EZH2 during EMT induction (60). EZH2 overexpression is linked to EMT induction and the migration of cancer cells, including those in pancreatic (61), NSCLC (62), and prostate cancer (63).

The PRC2 complex promotes LC progression by activating apoptosis and inhibiting the cell cycle. Dysregulation of the RB1/E2F pathway, in which the retinoblastoma protein (RB1) normally represses E2F transcription factors to inhibit proliferation, can lead to EZH2 overexpression in SCLC (64). Phosphorylation-mediated inactivation of RB1 can result in the release of the E2F protein, which acts as a transcription factor and triggers the activation of various factors involved in cell cycle progression, such as cyclins A and E. Overactivation of E2F can also induce p21 and activate apoptosis, leading to cell cycle inhibition (65, 66). As a downstream factor of the RB1/E2F pathway, dysregulation of this pathway can lead to the overexpression of EZH2 in SCLC (66). In addition, EZH2 exerts a negative influence on the expression of pro-apoptotic factors such as Puma and Bad, as well as cell cycle inhibitors like p21, ultimately promoting the development of SCLC tumors (66). LncRNAs, such as HOX transcript antisense intergenic RNA (HOTAIR), can affect PRC2 expression. For example, HOTAIR upregulation leads to EZH2 overexpression, which inhibits p21 and modulates CDK1 and the cell cycle at the G2/M transition (67). EZH2 overexpression also increases VEGF-A expression, activates AKT phosphorylation, and enhances proliferation, invasion, and migration of LC cells (68).

EZH2 can epigenetically suppress various negative regulators of the mTOR pathway, including TSC2, RHOA, and GPI, further inhibiting RHEB expression, thereby activating the mTOR signaling pathway and suppressing autophagy (69). A study has confirmed that enhanced protective autophagy could be one of the mechanisms leading to EGFR-TKI resistance in NSCLC, and knocking down or inhibiting EZH2 in NSCLC cells led to increased autophagy, indicating that EZH2 acts as a negative regulator of autophagy (70).

SUZ12 is an oncogene of NSCLC, and its knockdown inhibits tumor cell growth, migration, and invasion (71). Gargiulo et al. (72) discovered that EED deletion resulted in PRC2 inactivation and delayed tumor progression in KRAS-driven LC. RBBP4 is highly expressed in NSCLC tissues, and its knockout significantly reduces the proliferation and invasiveness of NSCLC cells, while its overexpression has the opposite effects (29).

## 6 PRC2 mediate therapy resistance in LC

Standard treatment options for LC includes surgery, platinum-based chemotherapy, radiotherapy, combination chemotherapy and targeted therapy, alone or in combination. However, treatment resistance and tumor progression remain major challenges for patients with advanced and metastatic LC. Therefore, researchers have increasingly focused on the potential of inhibitors of the PRC2 complex in tumor therapy due to the reversibility of epigenetic modifications. Studies have shown that EZH2 inhibition prevents the emergence of acquired resistance and enhances chemotherapeutic efficacy in LC.

Cancer stem cells (CSCs) are responsible for cancer progression and the induction of resistance to therapy. In LC cells, miRNA-21 promotes the expression level of EZH2 and enhances the progression of CSCs, leading to resistance to radiotherapy (73). EZH2 is also recruited through lncRNA 00665 to activate the PI3K/AKT pathway and induce acquired resistance to gefitinib in NSCLC (74). Furthermore, Riquelme et al. (75) observed that EZH2 expression can be induced by VEGF/VEGFR-2 activation through upregulation of E2F3 and hypoxia-inducible factor-1α (HIF1α), while downregulation of miR-101. They also found that depletion of EZH2 reduced the malignant potential of lung adenocarcinoma cells and increased their sensitivity to platinum-based or VEGFR-2 targeted therapies.

HOTAIR was found to be upregulated in gefitinib-resistant lung cancer cells, and its overexpression increased the resistance of LC cells to gefitinib. HOTAIR and EZH2 collaborated to silence the expression of p16 and p21 through H3K27me3 in the promoter regions, leading to accelerated gefitinib resistance. And inhibition of EZH2 sensitized LC cells to gefitinib by reversing the silencing of p16 and p21 mediated by HOTAIR and EZH2 (67).

## 7 Correlation between PRC2 expression and prognosis of LC patients

In a particular study, the research revealed an upregulation of EZH2 expression in NSCLC cells compared to normal human bronchial epithelial cells, as evidenced by protein blotting assay (76). Additionally, the study employed Taqman quantitative real-time RT-PCR to measure EZH2 expression in matched tumor and normal tissue samples. Notably, NSCLC patients whose tumors exhibited higher EZH2 expression experienced notably reduced overall survival rates, along with diminished disease-specific and disease-free survival rates, as compared to patients with lower EZH2 expression (*P* = 0.005, *P* = 0.001, and *P* = 0.003, respectively) (76). The study’s findings propose that EZH2 mRNA levels hold potential as a valuable prognostic indicator for NSCLC patients. In another study, 157 surgically resected cases of NSCLC were meticulously evaluated using immunohistochemistry. The study findings illuminated a compelling association between EZH2 expression in tumor cells and patient prognosis. Notably, patients with high EZH2 expression exhibited significantly poorer outcomes compared to those with low EZH2 expression across all pathological stages of NSCLC (*P* = 0.001), including the subset of patients with pathological stage I NSCLC (*P* = 0.006). Furthermore, a multifactorial analysis of this study underscored that high EZH2 expression independently served as a detrimental prognostic factor in patients with pathological stage I disease (*P* = 0.048). It’s worth noting that high EZH2 expression was notably linked with several clinicopathological characteristics, including non-adenocarcinoma histology (*P* = 0.001), moderate and low differentiation (*P* = 0.001), advanced pathological tumor classification (*P* = 0.02), and elevated Ki-67 and cell cycle protein E labeling index (*P* < 0.001) (77). They highlight the potential utility of EZH2 as an independent predictor of patient outcomes, particularly in the context of early-stage disease. Another study, confirmed through immunohistochemistry, established that EZH2 expression exhibited a significant increase in squamous lung carcinoma compared to adenocarcinoma. Within the adenocarcinoma subtype, higher EZH2 expression was notably associated with younger age, smoking history, and advanced TNM stage (78). These results shed light on the clinical significance of EZH2 in NSCLC and underscore its potential as a predictive biomarker for patient outcomes.

Furthermore, the deletion of EED has been demonstrated to promote the development of KRAS-driven NSCLCs (79). In a separate study, the expression levels of SUZ12 were assessed in 40 pairs of clinical NSCLC tissues and adjacent normal tissues using quantitative reverse transcription polymerase chain reaction (qRT-PCR). The findings from this study revealed significant dysregulation of SUZ12 expression in NSCLC tissues when compared to adjacent paracancerous tissues (*P*<0.05) (71). Moreover, this abnormal SUZ12 expression was closely associated with key clinical parameters, including tumor size, lymph node metastasis, and clinical stage (*P*<0.05) (71). Furthermore, the study conducted siRNA-mediated knockdown experiments of SUZ12, which demonstrated a noteworthy inhibition of tumor cell growth, migration, and invasion. These results strongly suggest a potential oncogenic role for SUZ12 in the initiation and progression of NSCLC. These compelling lines of evidence collectively indicate that SUZ12 functions as an oncogene in NSCLC. This implies that SUZ12 holds promise as a novel diagnostic marker for NSCLC, and it may emerge as a promising therapeutic target for interventions in NSCLC.

In summary, a compelling association emerges between the expression of the PRC2 complex and lung cancer prognosis, indicating its potential as a predictive biomarker for patient outcomes. Furthermore, techniques such as quantitative PCR and immunohistochemistry can be valuable tools in clinical studies for assessing the correlation between gene expression and various clinicopathological factors. Notably, quantitative PCR offers the advantage of simplicity and feasibility in such investigations.

## 8 PRC2 inhibitor

Given the pivotal role played by PRC2 in cancer development, it has become a potential therapeutic target for cancer. Different inhibitors with different mechanisms of action have been developed, including EZH2 inhibitors and EED inhibitors (**Table 1**).

**Table 1:** PRC2 inhibitors and their mechanisms of action.

Classification	Mechanism of action	Inhibitors/reference
**Enzymatic inhibition of EZH2**	Inhibiting the activity of SAH	DZNep (80)
	SAM-competitive inhibitors	GSK126 (8), GSK343 (8), GSK926 (81), tazemetostat (81), EPZ011989 (82), CPI-1205 (83), UNC1999 (8), JQEZ5 (8)
**Inhibitors that trigger EZH2 degradation**		FBW7, GNA022, ANCR, ZRAMB1 siRNA (84, 85)
**Inhibitors that disrupt PRC2 stability**	Disrupting the EZH2–EED complex	Astemizole, apomorphine hydrochloride, wedelolactone, oxyphenbutazone, nifedipine, AZD9291 (86), MAK683 (87)
	Disrupting the EZH2–SUZ12 interaction	A769662 (an AMPK agonist) (88)
**EED allosteric inhibitors**	Competitively inhibiting the binding of H3K27me3 to EED	EED162 (89), EED226 (90), MAK683 (91), EEDi-5285 (92), EEDi-1056 (92), EEDi-5273 (93), BR-001 (94), EED709 (95), A-395 (96), Jarid2114-118-K116 me3 (97), UNC5114 (97), UNC5115 (98),
**EED-targeted proteolysis-targeting chimeras (PROTACs)**	Impairing PRC2 protein function by ubiquitinating to induce proteasomal degradation of EED	UNC6852 (99), PROTAC 1 (100), PROTAC 2 (100)

EZH2 inhibitors can be categorized into three groups: enzymatic inhibitors, destabilizers of the PRC2 complex, and inducers of EZH2 degradation. Enzymatic inhibitors, such as 3-Deazaneplanocin A (DZNep), indirectly inhibit EZH2 by increasing the activity of S-adenosyl-L-homocysteine hydrolase (SAH), leading to apoptosis in tumor cells (101). However, DZNep is not specific for EZH2 and can globally inhibit histone methylation. Highly selective S-adenosyl-L-methionine (SAM)-competitive inhibitors of EZH2 methyltransferase activity, including tasimetostat and GSK126, have been developed and are undergoing clinical trials (102, 103). EZH2 inhibitors such as AZD9291 (86) and MAK683 (87, 98) reduce EZH2 protein levels by disrupting the EED-EZH2 interaction. Another strategy for inhibiting EZH2 is triggering EZH2 degradation, such as Gambogenic acid (GNA) derivative GNA022 (84).

Current cancer treatment strategies for EED include protein-protein interaction inhibitors, allosteric inhibitors, and proteolytically targeted chimeras (PROTACs). Compounds that competitively occupy the H3K27me3 binding pouch in EED can combat PRC2-driven carcinogenesis by inhibiting EED-H3K27me3 interactions and disrupting allosteric activation of PRC2 (104). EED allosteric inhibitors with therapeutic potential include EED162 (89), EED226 (90), MAK683 (91), BR-001 (105), EED709 (95), A-395 (96), Jarid2114-118-K116 me3 (97), UNC5114 (97), and UNC5115 (98). In EZH2-resistant cells, EZH2 inhibitors such as GSK126 and tazemetostat fail to inhibit the development of diffuse large B-cell lymphoma (DLBCL) cells, with a significant reduction of apoptosis (106). In contrast, EED inhibitors still show efficacy in suppressing the proliferation of these cells, suggesting the potential advantage of EED inhibitors for treating SAM-competitive EZH2 inhibitor-resistant cancers (94).

## 9 Current status of PRC2 inhibitors in LC treatment

We review the potential of PRC2-targeted inhibition in the treatment of LC, including its use in combination with other inhibitors and chemotherapeutic agents, as well as its impact on drug sensitivity. Additionally, we summarize ongoing clinical trials investigating various PRC2 inhibitors (**Table 2**).

**Table 2:** Current clinical trials of PRC2 inhibitors.

NCT number	Status	Drug	Conditions	First posted
NCT02860286	Completed	Tazemetostat	Mesothelioma BAP1 loss of function	2021
NCT04846478	Recruiting	Talazoparib; Tazemetostat	Metastatic prostate cancer Metastatic castration-resistant prostate cancer	2021
NCT04537715	Not yet recruiting	Tazemetostat Itraconazole Rifampin	Advanced malignancies Solid tumor Follicular lymphoma Non-Hodgkin lymphoma Diffuse large B-cell lymphoma (DLBCL) Synovial sarcoma Renal medullary carcinoma Mesothelioma Rhabdoid tumor	2020
NCT02860286	Completed	Tazemetostat	Mesothelioma BAP1 loss of function	2021
NCT04917042	Recruiting	Tazemetostat	Peripheral nerve sheath tumor	2021
NCT04241835	Recruiting	Tazemetostat	Hepatic impairment Advanced malignant solid tumor	2020
NCT04624113	Recruiting	Tazemetostat Pembrolizumab	Head and neck squamous cell carcinoma	2020
NCT05353439	Recruiting	Pembrolizumab Tazemetostat Hydrobromide Topotecan Hydrochloride	Extensive-stage and limited-stage lung small cell carcinoma Platinum-resistant and platinum-sensitive lung small cell carcinoma	2022
NCT04846478	Recruiting	Talazoparib Tazemetostat	Metastatic prostate cancer Metastatic castration-resistant prostate cancer	2021
NCT03854474	Recruiting	Pembrolizumab Tazemetostat	Locally advanced urothelial carcinoma metastatic urothelial carcinoma Stage IIIA - IVB bladder cancer AJCC v8	2019
NCT04557956	Recruiting	Dabrafenib Mesylate Tazemetostat Hydrobromide Trametinib Dimethyl Sulfoxide	Clinical stage IV cutaneous Melanoma AJCC v8 Metastatic melanoma	2020
NCT03028103	Completed	Tazemetostat Fluconazole Omeprazole Repaglinide	DLBCL Primary mediastinal lymphoma Mantle cell lymphoma Marginal zone lymphoma Advanced solid tumor	2021
NCT04179864	Recruiting	Tazemetostat Abiraterone/prednisone Enzalutamide	Metastatic prostate cancer	2019
NCT05151588	Not yet recruiting	Docetaxel Cis-platinum 5-FU Tazemetostat	Sinonasal carcinoma	2021
NCT05407441	Not yet recruiting	Tazemetostat Nivolumab Ipilimumab	Atypical teratoid rhabdoid tumor INI1 (SMARCB1)-deficient and SMARCA4-deficient primary CNS malignant tumors Chordoma Malignant rhabdoid tumor Rhabdoid tumor of the kidney Epithelioid arcoma	2022
NCT04705818	Recruiting	Durvalumab Tazemetostat	Advanced colorectal carcinoma Advanced soft-tissue sarcoma Advanced pancreatic adenocarcinoma	2021
NCT03480646	Active	CPI-1205 Enzalutamide Abiraterone/Prednisone	Metastatic castration-resistant prostate cancer	2018
NCT02900651	Recruiting	MAK683	DLBCL	2016

Tazemetostat is a selective oral EZH2 inhibitor that was approved in January 2020 as the first inhibitor to treat adults and adolescents who do not meet the indications for complete resection (in metastatic or locally advanced epithelial sarcoma) (102). Recent research shows that tazemetostat has a positive antitumor effect on solid tumors such as refractory malignant pleural mesothelioma (107), PBRM1-mutated human chordoma xenograft (108), and advanced epithelioid sarcoma with the loss of INI1/SMARCB1 (109). A clinical study evaluated the EZH2 inhibitor CPI-1205, which targets SAM, in patients with metastatic castration-resistant prostate cancer and is currently in early clinical trials (NCT03480646). Another clinical study, which is currently recruiting participants, is investigating the safety and efficacy of the EED inhibitor MAK683 in patients with various advanced malignancies, including gastric cancer, DLBCL, prostate cancer, nasopharyngeal carcinoma, ovarian cancer, and sarcoma (NCT02900651). In a particular study, it was observed that the growth of SCLC cell lines was inhibited when these cells were treated with EZH2 inhibitors, specifically DZNep and GSK126 (110). Additionally, when EZH2 was specifically targeted using DZNep, it was found to enhance the sensitivity of both SCLC cells and tumors to cisplatin (111). These findings underscore the potential therapeutic benefits of EZH2 inhibitors in the context of SCLC.

Although targeting EZH2 represents an attractive strategy for treating NSCLC, including those with BRG1 or EGFR mutations, targeting EZH2 alone is sometimes not effective in inhibiting the malignant proliferation of tumors (112–116). Therefore, combination therapies of chemotherapy and PRC2 inhibitors have been explored as a better strategy for treating cancer. Recent studies have designed and synthesized double EZH2/BRD4 inhibitors, which have shown significant antiproliferative activity and resensitization to EZH2 inhibitors in several solid cancers, including LC and pancreatic cancer, in xenograft mouse models (117). Furthermore, a multifunctional nanoparticle-based delivery system has been developed for efficient and safe co-delivery of EZH2 siRNA and etoposide to in orthotopic NSCLC (113). In this study, the combination of EZH2 siRNA and etoposide showed potent inhibition of tumor proliferation and metastasis in both *in vivo* and *in vitro* experiments.

While the experimental results of DZNep imply great clinical potential, its plasma half-life is very short, and it nonspecifically inhibits histone methylation, making it toxic in animal models (118). Therefore, there is an urgent need to develop relevant combination therapies. Our previous experimental results suggest that the combination of EZH2 inhibitors (GSK343 or DZNep) and gefitinib inhibits cell proliferation, migration, and tumor activity more effectively than either drug alone (119). This suggests that the combination of EZH2 inhibitors and epidermal growth factor receptor-tyrosine kinase inhibitors (EGFR-TKIs) is effective in treating patients with EGFR wild-type NSCLC who do not respond well to conventional EGFR-TKI therapy. Additionally, the combination of a double EZH2 inhibitor and a topoisomerase II inhibitor has been shown to have a therapeutic effect on EGFR mutant NSCLC and BRG1-mutant LC, which is resistant to standard chemotherapy (112). Additionally, there is a report that DZNep used in combination with the histone deacetylase inhibitor Norville can dramatically inhibit NSCLC cell proliferation and induce apoptosis (118). These results demonstrate that epigenetic-chemotherapy combination therapies have great potential to improve current oncology treatment regimens. A list of current clinical trials of PRC2 inhibitors is provided in **Table 2**.

## 10 Conclusion

The dysregulation of PRC2-mediated epigenetics has been related to the progression of LC, driving tumor proliferation, invasion, and metastasis. *In vitro* and *in vivo* studies have demonstrated that targeting EZH2 expression is effective in slowing LC progression and enhancing chemosensitivity. Several drugs that inhibit EZH2/PRC2 are under development and being evaluated in clinical trials, although most are still in preclinical or phase 1/2 studies. Research has shown that combining PRC2 inhibitors with other therapies, such as immunotherapy, chemotherapy, and targeted therapies, may achieve complementary or synergistic antitumor effects (120), and drug delivery methods such as nanocarriers may enhance therapeutic efficacy. Overall, PRC2 is a crucial epigenetic factor in the development and progression of LC, and PRC2 inhibitors show great promise for the treatment of cancer.

## Author contributions

MG, YL, and PC contributed equally to this work. All authors contributed to the article and approved the submitted version.
